# Supplementary material for: Single-Cell Sequencing Confirms Transcripts and VHDJH Rearrangements of Immunoglobulin Genes in Human Podocytes
Source: Genes (Basel). 2021 Mar 25;12(4):472. doi: 10.3390/genes12040472 (PMC8064494; doi:10.3390/genes12040472)
Supplement: Supplementary file 1 [file genes-12-00472-s001.zip › supplementary material/English-Editing-Certificate-27247.pdf]

We certify that the following article

# Single-cell sequencing confirms transcripts and VHDJH rearrangements of immunoglobulin genes in human podocytes

Zhenling Deng, Huige Yan, Zhan Shi, Xinyu Tian, Zhuan Cui, Yingchun Sun, Song Wang, Danxia Zheng, Xiaoyan Qiu *, Yue Wang *

has undergone English language editing by MDPI. The text has been checked for correct use of grammar and common technical terms, and edited to a level suitable for reporting research in a scholarly journal.
MDPI uses experienced, native English speaking editors. Full details of the editing service can be found at
► https://www.mdpi.com/authors/english.

MDPI
Multidisciplinary
Digital Publishing
Institute

Basel, Switzerland
February 2021

Dr. Shu-Kun Lin
Publisher & President
MDPI
