# Supplementary material for: Single-Cell Sequencing Confirms Transcripts and VHDJH Rearrangements of Immunoglobulin Genes in Human Podocytes
Source: Genes (Basel). 2021 Mar 25;12(4):472. doi: 10.3390/genes12040472 (PMC8064494; doi:10.3390/genes12040472)
Supplement: Supplementary file 1 [file genes-12-00472-s001.zip › supplementary material/revised supplementary material.docx]

Table SI. Clinicopathologic features of 10 patients used in this study.

| Case number | Age/Sex | Blood pressure (mmHg) | proteinuria (g/d) | Serum creatinine (μmol/L) | eGFR (ml/min/1.73m^2^) | Erythrocyturia (0 to 5+ ) |
| --- | --- | --- | --- | --- | --- | --- |
| MN1 | 28/M | 116/78 | 10 | 61 | 129 | 1 |
| MN2 | 49/M | 143/100 | 7.3 | 72 | 104 | 2 |
| MN3 | 30/M | 130/66 | 7.4 | 67 | 123 | 2 |
| IgAN1 | 30/F | 105/60 | 1.1 | 61 | 117 | 3 |
| IgAN2 | 29/F | 114/60 | 1.8 | 41 | 135 | 2 |
| IgAN3 | 24/F | 131/93 | 4.3 | 98 | 70 | 3 |
| IgAN4 | 41/F | 120/75 | 1.2 | 60 | 109 | 3 |
| IN | 32/M | 143/97 | 2.9 | 162 | 48 | 3 |
| HC | 38/M | 114/76 | ND | 82 | 105 | 3 |
| HC-10×G | 48/M | 130/86 | ND | 100 | 80 | 2 |
| Abbreviations: MN, membranous nephropathy; IgAN, immunoglobulin A nephropathy; IN, ischemic nephropathy; HC, healthy control; F, female; M, male; ND, not detected; HC-10×G, healthy control used for 10×Genomics Chromium system.  Table SII. Primers used in this study.   \| Gene \| Primer \| Primer sequence 5′-3′ \| \| --- \| --- \| --- \| \| Ig heavy chain constant region \| Antisense primer (IGHGc-R1) \| GAGTCCTGAGGACTGTAGGACAG \| \| Antisense primer (IGHAc-R1) \| TTCGCTCCAGGTCACACTGAG \| \| Antisense primer (IGHMc-R1) \| TGATGGAGTCGGGAAGGAAGTC \| \| Antisense primer (IGHDc-R1) \| GACAGTCACGGACGTTGGGT \| \| Antisense primer (IGHEc-R1) \| AGAGTCACGGAGGTGGCATTG \| \| Ig variable region (2^nd^ PCR) \| Internal sense primer (VH-FR2) \| TGGRTCCGVCAGSCYCCNGG \| \| Internal antisense primer (JH) \| AACTGCAGAGGAGACGGTGACC \| \| podocyte specific marker genes \| External sense primer (NPHS1) \| GGTGCAGTTTCCCCCAACTA \| \| External antisense primer (NPHS1) \| AGAAGGAGCTCACGGTTTCG \| \| Internal sense primer (NPHS1) \| CCGGGAGACGCCTTAAACTT \| \| Internal antisense primer (NPHS1) \| GAGCCTTTGAATGGGGCTCT \| \| External sense primer (NPHS2) \| GGTACCAAATCCTCCGGCTTA \| \| External antisense primer (NPHS2) \| CCTTTGCATCTTGGGCGATG \| \| Internal sense primer (NPHS2) \| GTGGCTTCTTGTCCTCATTTCC \| \| Internal antisense primer (NPHS2) \| CAGTGAGGGATCGATGTGCT \| \| B cell  marker gene \| External sense primer (CD19) \| GAGGGAGATAGGCACGGATGG \| \| External antisense primer (CD19) \| AACATTGCTCCAGAGGTTGGC \| \| Internal sense primer (CD19) \| GTGGCTACTGGCTTTCAGG \| \| Internal antisense primer (CD19) \| CAGGTGTGAATCTTGGGGACTT \| | | | | | | |

Table SIII. The detection rate of Ig^+^ single podocytes and five classes of Ig heavy chains.

| Case | No.of single cells | Ig^+^single podocytes（ratio） | podocyte-derived Ig class(ratio) | | | | |
| --- | --- | --- | --- | --- | --- | --- | --- |
|  |  |  | IgG | IgM | IgA | IgE | IgD |
| HC | 32 | 6/32 (18.8%) | 3/6 （50%） | 3/6（50%） | 0 | 0 | 0 |
| IgAN | 120 | 15/120 (12.5%) | 14/15 (93.3%) | 5/15 (33.3%) | 1/15 (6.7%) | 0 | 0 |
| MN | 60 | 18/60 (30%) | 9/18 (50%) | 15/18 (83.3%) | 3/18 (16.7%) | 0 | 0 |
| IN | 23 | 9/23 (39.1%) | 3/9 (33.3%) | 4/9 (44.4%) | 2/9 (22.2%) | 5/9 (55.6%) | 2/9 (22.2%) |
| Total | 235 | 48/235 (20.4%) | 29/48 (60.4%) | 27/48 (56.3%) | 6/48 (12.5%) | 5/48 (10.4%) | 2/48 (4.2%) |

Table SIV. Rate of functional rearrangement in 429 V_H_DJ_H_ rearrangements.

| sample origin | Ig class | No. of single cell | No. of clone | No. of functional V_H_DJ_H_ rearrangement | No. of non-functional V_H_DJ_H_ rearrangement (%) |
| --- | --- | --- | --- | --- | --- |
| HC | γ chain | 3 | 20 | 20 | 0 |
|  | μ chain | 3 | 17 | 17 | 0 |
| IgAN | α chain | 1 | 5 | 5 | 0 |
|  | γ chain | 14 | 75 | 68 | 7 (9.3%) |
|  | μ chain | 5 | 28 | 26 | 2 (7.1%) |
| MN | α chain | 3 | 27 | 26 | 1 (3.7%) |
|  | γ chain | 9 | 66 | 65 | 1 (1.5%) |
|  | μ chain | 15 | 83 | 83 | 0 |
| IN | α chain | 2 | 13 | 13 | 0 |
|  | γ chain | 5 | 24 | 19 | 5 (20.8%) |
|  | μ chain | 4 | 25 | 24 | 1 (4%) |
|  | δ chain | 2 | 14 | 14 | 0 |
|  | ε chain | 5 | 32 | 31 | 1 (3.1%) |
| total |  | 71 | 429 | 411 | 18 (4.2%) |

Abbreviations: HC, healthy control; IgAN, immunoglobulin A nephropathy; MN, membranous nephropathy; IN, ischemic nephropathy

Table SV. The V_H_DJ_H_ rearrangement patterns of Ig heavy chains variable region in 48 single podocytes.

| Cases No. | Cell No. | Ig type | V_H_DJ_H_ recombination | CDR3 sequence | Clones |
| --- | --- | --- | --- | --- | --- |
| HC | HC-1 | μ chain | IGHV4-39/IGHD1-1/IGHJ3 | GCGAGACACCAGATCATTCCCCAACTGGGTGATGCTTTTGATATC | 7/7 |
|  | HC-2 | γ chain | IGHV1-2/IGHD3-3/IGHJ6 | GCGAGAGGGGGTCATAGGTACGATGATTACTATTACTACTACGGTATGGACGTC | 6/6 |
|  | HC-3 | γ chain | IGHV5-51/IGHD3-16/IGHJ4 | GCGAGATGGAATGTTATGATTGGATTTTATACAGCTGACTAC | 1/9 |
|  |  |  | IGHV7-4/IGHD3-10/IGHJ4 | GCGGTGGGGGTAGCCTATGGTTCGGGGAGCTCCTTTGACTAC | 8/9 |
|  | HC-4 | μ chain | IGHV3-7/IGHD2-8/IGHJ4 | ACGGGACAGCCGAGAATGCTGGCCCAC | 1/5 |
|  |  |  | IGHV3-7/IGHD1-1/IGHJ4 | ACGGGACAGCCGAGAACGCTGGCCCAC | 4/5 |
|  | HC-5 | γ chain | IGHV1-24/IGHD2-15/IGHJ4 | GCAACTTGTGATGGTAGCTGCTACTCACCCGATCCGACCTTTCACTTC | 5/5 |
|  | HC-6 | μ chain | IGHV1-18/IGHD2-2/IGHJ6 | GCGAGAGATCTGGATATTGTAGTAGTAGCAGCTGCTACTCGCTACTACGGTTTGGACGTC | 5/5 |
| IgAN1 | IgAN1-1 | γ chain | IGHV3-48/IGHD6-19/IGHJ5 | GCGAGAGGGTATAAAAGTGGCTACTATGGTGCC | 11/11 |
| IgAN2 | IgAN2-1 | γ chain | IGHV1-24/IGHD2-15/IGHJ4 | GCAACTTGTGATGGTAGCTGCTACTCACCCGATCCGACCTTTCACTTC | 4/4 |
|  | IgAN2-2 | γ chain | IGHV3-48/IGHD3-3/IGHJ4 | GCGAGATCGTCAAGAGGAGTGGGCATTGACTAT | 5/5 |
|  |  | μ chain | IGHV3-74/IGHD5-18/IGHJ4 | GCAAGAGCCCCCCCGGGTAGTTTCACTGGATACCATTTCGACTAC | 4/6 |
|  |  |  | IGHV3-23/IGHD6-13/IGHJ2 | GCGAAAGCTGTGGCAGCAGATGGTCTCTGGTACTTCGATCTC | 2/6 |
|  | IgAN2-3 | γ chain | IGHV1-18/IGHD6-19/IGHJ5 | GCGAGAGATGATTCGTCCACAGGGTATCTCAGTGGCTGGTCTTCGTCCAACTGGCTCGACCCC | 1/5 |
|  |  |  | IGHV1-24/IGHD2-2/IGHJ3 | GCAACATCGGATCCTGAGATACTTCCAGCTGCTCGCTGGGGTGAGGCTTTTGATGTC | 2/5 |
|  |  |  | IGHV3-23/IGHD1-14/IGHJ4 | GTAAAACAAAAGTACTTTGATTCGACGAATAAGAATAACCGAGTTTTTGATGTC | 2/5 |
|  | IgAN2-4 | γ chain | IGHV3-53/IGHD3-10/IGHJ6 | GCCAGAGATCGTTACTACTTTGGTTCGGGGAGTTACCCCCTGGCCCTCTTAGCTATGGACGTC | 3/3 |
|  | IgAN2-5 | γ chain | IGHV1-8/IGHD1-1/IGHJ4 | GCGAGAGGCCGCGGCCTAAGCGCAACTGGTACGAGTAGGTTCGTTCTGGCCTTC | 5/5 |
|  | IgAN2-6 | α chain | IGHV3-23/IGHD6-13/IGHJ4 | GCGAAAGCGATGAGCAGCAGCAGCCGATACGTCTTTGACTAC | 5/5 |
|  | IgAN2-7 | γ chain | IGHV4-39/IGHD3-16/IGHJ4 | GCGAGACACTCGGAAAGGGGGAGTTCATACTTTGACTAC | 4/4 |

| Cases No. | Cell No. | Ig type | V_H_DJ_H_ recombination | CDR3 sequence | Clones |
| --- | --- | --- | --- | --- | --- |
| IgAN2 | IgAN2-8 | γ chain | IGHV3-23/IGHD6-13/IGHJ4 | GCGAAACCTCATAGTAGCAGCTCTGGTTTTGACTAC | 1/3 |
|  |  |  | IGHV1-8/IGHD5-12/IGHJ4 | GCGAGGGGCTCCAGACTTAGTGGCCACGATGCGGCCGGGACATTTGACTAC | 1/3 |
|  |  |  | IGHV7-4-1/IGHD5-12/IGHJ5 | GCGAGGACCACGATTACCAACAGCTGGGCGAGGTGGTTCGACCCC | 1/3 |
|  |  | μ chain | IGHV1-18/IGHD3-3/IGHJ6 | GCGAGAGATCGACAGTACGATTTTTGGAGTGGTTCCTACTACTACTACTACATGGACGTC | 3/4 |
|  |  |  | IGHV4-34/IGHD3-10/IGHJ6 | GCGGGAACGGGGAGGCGCTACTACTACGGTATGGACGTC | 1/4 |
|  | IgAN2-9 | γ chain | IGHV4-59/IGHD3-22/IGHJ3 | GCGGGGGATCGTTCATACTATGATAGCCGGAGTTTTTACCCTGATGCTCTTGATATC | 5/5 |
|  |  | μ chain | IGHV3-53/IGHD6-19/IGHJ4 | ACTGGAGATGATGGAACGTATAGCAGTGGCTGGTACCACGGCTAC | 4/4 |
|  | IgAN2-10 | γ chain | IGHV4-59/IGHD3-22/IGHJ3 | GCGAGGGATCGTTCATACTATGATAGCCGGAGTTTTTACCCTGATGCTCTTGATATC | 5/5 |
| IgAN3 | IgAN3-1 | γ chain | IGHV5-51/IGHD3-16/IGHJ4 | GCGGGATGGAATGTTATGATTGGATTTTATACAGCTGACTAC | 2/2 |
|  |  | μ chain | IGHV3-7/IGHD2-21/IGHJ4 | GCGAGGAGTGATTGGGGGCCTGACTAC | 3/8 |
|  |  |  | IGHV3-74/IGHD5-18/IGHJ4 | GCAAGAGCCCCCCCGGGTAGTTTCACTGGATACCATTTCGACTAC | 1/8 |
|  |  |  | IGHV4-61/IGHD2-21/IGHJ5 | GCGAGAGCGGATTGTGGTCGTGCTGGCTGTTACACCCCATTTTACAACTGGTTCGACCCC | 4/8 |
|  | IgAN3-2 | γ chain | IGHV3-23/IGHD6-13/IGHJ1 | GCGAAAGACCGTGGGCGAATAGGAGCACCTGGTACTCTCGTCGTCGCCGTGAAACCCTCTGA  ATACTTCCAACAT | 6/6 |
| IgAN4 | IgAN4-1 | γ chain | IGHV1-18/IGHD3-9/IGHJ6 | GCGAGAGTCTTAGTGGTTCACGATCCTTTGACTGGTTACCACTACAGTATGGACGTC | 5/5 |
|  | IgAN4-2 | μ chain | IGHV3-23/IGHD3-3/IGHJ5 | GCGAACATAATGTATTGGCAGCCTGAATCC | 1/4 |
|  |  |  | IGHV3-7/IGHD3-3/IGHJ5 | GCGAACATAATGTATTGGCAGCCTGAATCC | 3/4 |
|  |  | γ chain | IGHV1-8/IGHD3-3/IGHJ5 | GCGAGAGGCAACCGGGGCCAGCATACTGCGATTTTTGGAGTGGTTATCCTATGGTGGTTCGA  CCCC | 5/5 |

| Cases No. | Cell No. | Ig type | V_H_DJ_H_ recombination | CDR3 sequence | Clones |
| --- | --- | --- | --- | --- | --- |
| MN1 | MN1-1 | γ chain | IGHV1-69/IGHD3-3/IGHJ4 | GCGAGAGTTCGAGGGGGGCGTATAACGATTTTTGGAGGGCTTGAGTAC | 2/5 |
|  |  |  | IGHV3-15/IGHD2-8/IGHJ5 | ACCACAGGATATTGTACTGATATTAGGTGCGTCACC | 3/5 |
|  |  | μ chain | IGHV1-18/IGHD5-12/IGHJ5 | GCGAGCACCAATAGTGGCTACGATGGGGGGCTTGGTCCC | 5/5 |
|  | MN1-2 | γ chain | IGHV1-18/IGHD1-1/IGHJ4 | GCGAGGGACAACTTTATTTCGCCCACTGAAGGTCCCTTTGACAAC | 7/7 |
|  |  | μ chain | IGHV1-18/IGHD6-19/IGHJ4 | GCGAGAGACCTGGTTCGCGAAATAGCAGTGGCTGGTACGACGAGGAGGGGGGGCTTTGACTAC | 2/2 |
|  | MN1-3 | γ chain | IGHV1-18/IGHD3-3/IGHJ6 | GCGAGAGTCTTACAGGTTCACGATCCCTTGAGTGGTTACCACTGCAATATGGACGTC | 10/10 |
|  | MN1-4 | α chain | IGHV4-34/IGHD3-3/IGHJ1 | GCGAGTCCAGACGGGAGTGCTTATGCCTTCCTCTAC | 1/6 |
|  |  |  | IGHV3-7/IGHD3-10/IGHJ5 | GTACTGTGGTTCGACATCGGAGGGAACCAC | 5/6 |
|  |  | γ chain | IGHV1-46/N/A/IGHJ6 | GCGAGATCCAAGAACGGAATGGACGTC | 5/5 |
|  |  | μ chain | IGHV3-69/IGHD3-22/IGHJ5 | GTGGGAGGATGCTATAGTAGTGGTTATTACTGGTTCGACCCC | 6/6 |
|  | MN1-5 | μ chain | IGHV3-48/IGHD2-15/IGHJ4 | GCGCGAGATTTAGGATATTGTAGTGGTGGTCGCTGCTCCCTTATTTACCACTTTGACTCC | 3/6 |
|  |  |  | IGHV4-34/IGHD6-13/IGHJ4 | GCGAGGCTGTATAGCAGCAGCTGC | 3/6 |
|  | MN1-6 | γ chain | IGHV3-53/N/A/IGHJ6 | GCGAGATCCATGGACGTC | 8/8 |
|  |  | μ chain | IGHV1-3/IGHD6-25/IGHJ4 | GCGACTGTAGCAGCGTCCTTGGGGGGCTAC | 7/7 |
|  | MN1-7 | μ chain | IGHV1-18/IGHD6-19/IGHJ4 | GCGAGAGACTCAGAAATAGCAGTGGCTGGTACAGGCGACTAC | 6/6 |
|  | MN1-8 | μ chain | IGHV5-51/IGHD2-15/IGHJ6 | GCGAGATATGGTCTGCGGGGCTGTAGTGATAAATGTTATGTTAGTTTCTACTACTACGGTATG  GACGTC | 4/4 |
| MN2 | MN2-1 | γ chain | IGHV3-11/IGHD4-23/IGHJ6 | GCGAGACTTCCTCGTGAGGTGGGAACTCCGTACTACTTCTACGGTATGGACGTC | 5/5 |
|  |  | μ chain | IGHV3-15/IGHD6-13/IGHJ4 | ACTTGGTCAAGCACCTGGTCACTCAGGTTCTACTTTGACTCC | 4/8 |
|  |  |  | IGHV3-64/IGHD5-12/IGHJ4 | GCGAGAGGGGGCCGGGTGGTGGCTACGGAGGCCGGCTAC | 2/8 |
|  |  |  | IGHV4-59/IGHD2-21/IGHJ4 | GCGAGAGGGGGGGAAGTGGTGACGACTAGCCCCTCTCACTTTGACTAC | 1/8 |
|  |  |  | IGHV1-18/IGHD2-2/IGHJ4 | GCGAGAGACGACGCGGATGGGTTGGACGAATCAGCTTCAATTGACTAC | 1/8 |

| Cases No. | Cell No. | Ig type | V_H_DJ_H_ recombination | CDR3 sequence | Clones |
| --- | --- | --- | --- | --- | --- |
| MN2 | MN2-2 | μ chain | IGHV1-18/IGHD3-22/IGHJ4 | GCGAGAACCCTTAGGCGTGAGAGTAGTGGTTATTCCCTATAC | 6/6 |
|  | MN2-3 | α chain | IGHV3-23/IGHD3-16/IGHJ4 | GTGAAATATGGAGATTTCCTTTGGGGGGGTTATCGTTTGAACTACTTTGACTAC | 6/6 |
|  |  | μ chain | IGHV3-21/IGHD3-10/IGHJ4 | GCGAGGTATTACTATGGTTCGTGGACTTATTATAACACCTACTTTGACTAT | 4/4 |
|  | MN2-4 | μ chain | IGHV1-18/IGHD1-26/IGHJ5 | GCGAGAGATCGGAGTGGGAGCTACCCCCCCGCTTGGTGGTTCGACCCC | 1/6 |
|  |  |  | IGHV3-23/IGHD3-10/IGHJ4 | GCGAAAGGAGGGTATTACCATGGTTCGGGGAACTACTTTGACTTC | 5/6 |
|  | MN2-5 | μ chain | IGHV1-18/IGHD3-22/IGHJ4 | GCAAGACTTCAGAGTGATAGTAGTGGTTATTACCACCAACCT | 6/6 |
|  | MN2-6 | μ chain | IGHV3-9/IGHD5-24/IGHJ4 | GCAAAAGTGGGGTATGGCTACAATTACTGGGGGCACTTTGACTAC | 5/5 |
|  | MN2-7 | μ chain | IGHV1-18/IGHD3-22/IGHJ4 | GCGAGAGATCATGATAGTAGTGGTTATTACGAACGGTTCTTTGACTAC | 4/6 |
|  |  |  | IGHV1-58/IGHD2-15/IGHJ6 | GTGGCAGGACCATATTGTAGTGGTGGTAGCTACTACGACTCCAATTACTACTACTACTACGGTAT  GGACGTC | 2/6 |
|  | MN2-8 | γ chain | IGHV1-2/IGHD1-1/IGHJ5 | GCGAGAGACCCTGAAGCGGGTTTGGAAGCAACTGATAACTGGTTCGACCCC | 3/3 |
|  |  | μ chain | IGHV3-15/IGHD3-16/IGHJ4 | GTTTGGTCAAGCACATGGTCAACTCGGTTCTACCTTGACCGC | 5/6 |
|  |  |  | IGHV4-30/IGHD6-13/IGHJ4 | GCGCTAGCGGAAGGCAGCAGCTGGTATCTCTACGACTAC | 1/6 |
| MN3 | MN3-1 | α chain | IGHV3-23/IGHD3-10/IGHJ3 | GCGAGAGTCACGGAGTTTCACACTCCGCCCTCCTTTCGGGGGGATGCTTTTGATCTC | 14/14 |
|  |  | γ chain | IGHV1-2/IGHD3-16/IGHJ6 | GCGAGAGGGGGTCATAGGTATGATGATTACTATTACTACTACGGTATGGACGTC | 12/12 |
|  | MN3-2 | γ chain | IGHV1-69/IGHD2-15/IGHJ4 | GCGACCCTAAAGGGATATTGCGGCTTTGGAAGCTGCTACTCGTTCTTTCTTGACGGCCCTGACAAC | 10/10 |
| IN | IN-1 | μ chain | IGHV4-59/IGHD3-22/IGHJ3 | GCGAGAGATGTAACGTATGATAGTAGTGGTTATAATGCTTTTGATATC | 5/5 |
|  | IN-2 | ε chain | IGHV3-23/IGHD2-21/IGHJ4 | GCGCGAAGGGTGACGGACTTGGGGATCCGTCAG | 5/5 |
|  | IN-3 | ε chain | IGHV4-59/IGHD3-22/IGHJ3 | GCGAGGGATCGTTCATACTATGATAGCCGGAGTCTTTACCCTGATGCTCTTGATATC | 1/6 |
|  |  |  | IGHV3-30/IGHD2-2/IGHJ6 | GCGAAAACGAAGGGTTGTAGTCGTACCACTTGCTACTATTACGGAATGGACATC | 5/6 |

| Cases No. | Cell No. | Ig type | V_H_DJ_H_ recombination | CDR3 sequence | Clones |
| --- | --- | --- | --- | --- | --- |
| IN | IN-4 | γ chain | IGHV3-9/IGHD3-10/IGHJ4 | GCAAAAGATCTCGCCCCAACGGGGATGCTTCGGGGGCCATTTGATAAC | 4/4 |
|  |  | μ chain | IGHV3-7/IGHD1-26/IGHJ6 | GCGAGAATCGGGGGTGGGAGGTACGATGGGTACTACTACGGTCTGGACGTC | 9/11 |
|  |  |  | IGHV1-18/IGHD5-12/IGHJ4 | GCGAGATCCCTCTCATATGGTGGCTACGATTTCGCTTAC | 2/11 |
|  |  | ε chain | IGHV3-9/IGHD3-10/IGHJ4 | GCAAAAGATCTCGCCCCAACGGGGATGCTTCGGGGGCCATTTGATAAC | 8/8 |
|  | IN-5 | α chain | IGHV4-59/IGHD5-21/IGHJ6 | GCGAGAGATAGGCATTATGGTGGCTACGATCGGGACTACTACTACTACATGGACGTC | 5/6 |
|  |  |  | IGHV3-30/IGHD3-10/IGHJ4 | GCGAGAGCGTATTACTATGGTTCGGGGAGTTATTATTACTTTGACTAC | 1/6 |
|  |  | μ chain | IGHV4-59/IGHD5-21/IGHJ6 | GCGAGAGATAGGCATTATGGTGGCTACGATCGGGACTACTACTACTACATGGACGTC | 1/3 |
|  |  |  | IGHV3-30/IGHD3-10/IGHJ4 | GCGAGAGCGTATTACTATGGTTCGGGGAGTTATTATTACTTTGACTAC | 1/3 |
|  |  |  | IGHV4-34/IGHD3-10/IGHJ6 | GCGAGAGGGCTTGAAGGCGGGGCGCACTACTACTACATGGACGTC | 1/3 |
|  |  | δ chain | IGHV4-59/IGHD5-21/IGHJ6 | GCGAGAGATAGGCATTATGGTGGCTACGATCGGGACTACTACTACTACATGGACGTC | 6/9 |
|  |  |  | IGHV4-34/IGHD3-10/IGHJ6 | GCGAGAGGGCTTGAAGGCGGGGCGCACTACTACTACATGGACGTC | 2/9 |
|  |  |  | IGHV3-30/IGHD3-10/IGHJ4 | GCGAGAGCGTATTACTATGGTTCGGGGAGTTATTATTACTTTGACTAC | 1/9 |
|  |  | ε chain | IGHV3-23/IGHD2-21/IGHJ4 | GCGCGAAGGGTGACGGACTTGGGGATCCGTCAG | 7/7 |
|  | IN-6 | α chain | IGHV1-24/IGHD5-18/IGHJ1 | TCTATTGGGGCTATGGCCACC | 7/7 |
|  |  | γ chain | IGHV1-69/IGHD2-15/IGHJ4 | GCGACCCTAAAGGGATATTGTGGCTTTGGAAGCTGCTACTCGTTCTTTCCTGACGGCCCTGACAAC | 10/10 |
|  | IN-7 | δ chain | IGHV4-34/IGHD2-2/IGHJ6 | GCGAGAATTGTAGTAGTACCAGCTGCCTTCGGAAGAGGTTATTACTACTACTACTACGGTATGGAC  GTC | 5/5 |
|  | IN-8 | γ chain | IGHV3-7/NA/IGHJ6 | GTGGGGGCCTACGGTATGGACGTC | 5/5 |
|  | IN-9 | μ chain | IGHV4-34/IGHD3-22/IGHJ4 | GCGAGAGGCCGGTCGTATGATAATAGTGGTTATCCAAACCCCTTGGACTAC | 5/5 |
|  |  | ε chain | IGHV3-23/IGHD2-21/IGHJ4 | GCGCGAAGGGTGACGGACTTGGGGATCCGTCAG | 5/5 |
