# Supplementary material for: Single-Cell Sequencing Confirms Transcripts and VHDJH Rearrangements of Immunoglobulin Genes in Human Podocytes
Source: Genes (Basel). 2021 Mar 25;12(4):472. doi: 10.3390/genes12040472 (PMC8064494; doi:10.3390/genes12040472)
Supplement: Supplementary file 1 [file genes-12-00472-s001.zip › supplementary material/revised supplementary material.pdf]

Table SI. Clinicopathologic features of 10 patients used in this study.

Case number	Age/Sex	Blood pressure (mmHg)	proteinuria (g/d)	Serum creatinine (μmol/L)	eGFR (ml/min/1.73m^2)	Erythrocyturia (0 to 5+)
MN1	28/M	116/78	10	61	129	1
MN2	49/M	143/100	7.3	72	104	2
MN3	30/M	130/66	7.4	67	123	2
IgAN1	30/F	105/60	1.1	61	117	3
IgAN2	29/F	114/60	1.8	41	135	2
IgAN3	24/F	131/93	4.3	98	70	3
IgAN4	41/F	120/75	1.2	60	109	3
IN	32/M	143/97	2.9	162	48	3
HC	38/M	114/76	ND	82	105	3
HC-10×G	48/M	130/86	ND	100	80	2

Abbreviations: MN, membranous nephropathy; IgAN, immunoglobulin A nephropathy; IN, ischemic nephropathy; HC, healthy control; F, female; M, male; ND, not detected; HC-10×G, healthy control used for 10×Genomics Chromium system.

Table SII. Primers used in this study.

Gene	Primer	Primer sequence 5′-3′
Ig heavy chain constant region	Antisense primer (IGHGc-R1)	GAGTCCTGAGGACTGTAGGACAG
	Antisense primer (IGHAc-R1)	TTCGCTCCAGGTCACACTGAG
	Antisense primer (IGHMc-R1)	TGATGGAGTCGGGAAGGAAGTC
	Antisense primer (IGHDc-R1)	GACAGTCACGGACGTTGGGT
	Antisense primer (IGHEc-R1)	AGAGTCACGGAGGTGGCATTG
Ig variable region (2nd PCR)	Internal sense primer (VH-FR2)	TGGRTCCGVCAGSCYCCNGG
	Internal antisense primer (JH)	AACTGCAGAGGAGACGGTGACC
podocyte specific marker genes	External sense primer (NPHS1)	GGTGCAGTTTCCCCCAACTA
	External antisense primer (NPHS1)	AGAAGGAGCTCACGGTTTCG
	Internal sense primer (NPHS1)	CCGGGAGACGCCTTAAACTT
	Internal antisense primer (NPHS1)	GAGCCTTTGAATGGGGCTCT
	External sense primer (NPHS2)	GGTACCAAATCCTCCGGCTTA
	External antisense primer (NPHS2)	CCTTTGCATCTTGGGCGATG
	Internal sense primer (NPHS2)	GTGGCTTCTTGTCCTCATTTCC
	Internal antisense primer (NPHS2)	CAGTGAGGGATCGATGTGCT
B cell marker gene	External sense primer (CD19)	GAGGGAGATAGGCACGGATGG
	External antisense primer (CD19)	AACATTGCTCCAGAGGTTGGC
	Internal sense primer (CD19)	GTGGCTACTGGCTTTCAGG
	Internal antisense primer (CD19)	CAGGTGTGAATCTTGGGGACTT

Table SIII. The detection rate of Ig^+ single podocytes and five classes of Ig heavy chains.

Case	No.of single cells	Ig^+single podocytes（ratio）	podocyte-derived Ig class(ratio)				
			IgG	IgM	IgA	IgE	IgD
HC	32	6/32 (18.8%)	3/6 （50%）	3/6 （50%）	0	0	0
IgAN	120	15/120 (12.5%)	14/15 (93.3%)	5/15 (33.3%)	1/15 (6.7%)	0	0
MN	60	18/60 (30%)	9/18 (50%)	15/18 (83.3%)	3/18 (16.7%)	0	0
IN	23	9/23 (39.1%)	3/9 (33.3%)	4/9 (44.4%)	2/9 (22.2%)	5/9 (55.6%)	2/9 (22.2%)
Total	235	48/235 (20.4%)	29/48 (60.4%)	27/48 (56.3%)	6/48 (12.5%)	5/48 (10.4%)	2/48 (4.2%)

Table SIV. Rate of functional rearrangement in 429 V_HDJ_H rearrangements.

sample origin	Ig class	No. of single cell	No. of clone	No. of functional V_HDJ_H rearrangement	No. of non-functional V_HDJ_H rearrangement (%)
HC	γ chain	3	20	20	0
	μ chain	3	17	17	0
IgAN	α chain	1	5	5	0
	γ chain	14	75	68	7 (9.3%)
	μ chain	5	28	26	2 (7.1%)
MN	α chain	3	27	26	1 (3.7%)
	γ chain	9	66	65	1 (1.5%)
	μ chain	15	83	83	0
IN	α chain	2	13	13	0
	γ chain	5	24	19	5 (20.8%)
	μ chain	4	25	24	1 (4%)
	δ chain	2	14	14	0
	ε chain	5	32	31	1 (3.1%)
total		71	429	411	18 (4.2%)

Abbreviations: HC, healthy control; IgAN, immunoglobulin A nephropathy; MN, membranous nephropathy; IN, ischemic nephropathy

Table SV. The V_HDJ_H rearrangement patterns of Ig heavy chains variable region in 48 single podocytes.

Cases No.	Cell No.	Ig type	V_HDJ_H recombination	CDR3 sequence	Clones
HC	HC-1	μ chain	IGHV4-39/IGHD1-1/IGHJ3	GCGAGACACCAGATCATTCCCCAACTGGGTGATGCTTTTGATATC	7/7
	HC-2	γ chain	IGHV1-2/IGHD3-3/IGHJ6	GCGAGAGGGGGTCATAGGTACGATGATTACTATTACTACTACGGTATGGACGTC	6/6
	HC-3	γ chain	IGHV5-51/IGHD3-16/IGHJ4	GCGAGATGGAATGTTATGATTGGATTTTATACAGCTGACTAC	1/9
			IGHV7-4/IGHD3-10/IGHJ4	GCGGTGGGGGTAGCCTATGGTTCGGGGAGCTCCTTTGACTAC	8/9
	HC-4	μ chain	IGHV3-7/IGHD2-8/IGHJ4	ACGGGACAGCCGAGAATGCTGGCCCAC	1/5
			IGHV3-7/IGHD1-1/IGHJ4	ACGGGACAGCCGAGAACGCTGGCCCAC	4/5
	HC-5	γ chain	IGHV1-24/IGHD2-15/IGHJ4	GCAACTTGTGATGGTAGCTGCTACTCACCCGATCCGACCTTTCACTTC	5/5
	HC-6	μ chain	IGHV1-18/IGHD2-2/IGHJ6	GCGAGAGATCTGGATATTGTAGTAGTAGCAGCTGCTACTCGCTACTACGGTTTGGACGTC	5/5
IgAN1	IgAN1-1	γ chain	IGHV3-48/IGHD6-19/IGHJ5	GCGAGAGGGTATAAAAGTGGCTACTATGGTGCC	11/11
IgAN2	IgAN2-1	γ chain	IGHV1-24/IGHD2-15/IGHJ4	GCAACTTGTGATGGTAGCTGCTACTCACCCGATCCGACCTTTCACTTC	4/4
	IgAN2-2	γ chain	IGHV3-48/IGHD3-3/IGHJ4	GCGAGATCGTCAAGAGGAGTGGGCATTGACTAT	5/5
		μ chain	IGHV3-74/IGHD5-18/IGHJ4	GCAAGAGCCCCCCCGGGTAGTTTCACTGGATACCATTTCGACTAC	4/6
			IGHV3-23/IGHD6-13/IGHJ2	GCGAAAGCTGTGGCAGCAGATGGTCTCTGGTACTTCGATCTC	2/6
	IgAN2-3	γ chain	IGHV1-18/IGHD6-19/IGHJ5	GCGAGAGATGATTCGTCCACAGGGTATCTCAGTGGCTGGTCTTCGTCCAACTGGCTCGACCCC	1/5
			IGHV1-24/IGHD2-2/IGHJ3	GCAACATCGGATCCTGAGATACTTCCAGCTGCTCGCTGGGGTGAGGCTTTTGATGTC	2/5
			IGHV3-23/IGHD1-14/IGHJ4	GTAAAACAAAAGTACTTTGATTCGACGAATAAGAATAACCGAGTTTTTGATGTC	2/5
	IgAN2-4	γ chain	IGHV3-53/IGHD3-10/IGHJ6	GCCAGAGATCGTTACTACTTTGGTTCGGGGAGTTACCCCCTGGCCCTCTTAGCTATGGACGTC	3/3
	IgAN2-5	γ chain	IGHV1-8/IGHD1-1/IGHJ4	GCGAGAGGCCGCGGCCTAAGCGCAACTGGTACGAGTAGGTTCGTTCTGGCCTTC	5/5
	IgAN2-6	α chain	IGHV3-23/IGHD6-13/IGHJ4	GCGAAAGCGATGAGCAGCAGCAGCCGATACGTCTTTGACTAC	5/5
	IgAN2-7	γ chain	IGHV4-39/IGHD3-16/IGHJ4	GCGAGACACTCGGAAAGGGGGAGTTCATACTTTGACTAC	4/4

Cases No.	Cell No.	Ig type	V_HDJ_H recombination	CDR3 sequence	Clones
IgAN2	IgAN2-8	γ chain	IGHV3-23/IGHD6-13/IGHJ4	GCGAAACCTCATAGTAGCAGCTCTGGTTTTGACTAC	1/3
			IGHV1-8/IGHD5-12/IGHJ4	GCGAGGGGCTCCAGACTTAGTGGCCACGATGCGGCCGGGACATTTGACTAC	1/3
			IGHV7-4-1/IGHD5-12/IGHJ5	GCGAGGACCACGATTACCAACAGCTGGGCGAGGTGGTTCGACCCC	1/3
		μ chain	IGHV1-18/IGHD3-3/IGHJ6	GCGAGAGATCGACAGTACGATTTTTGGAGTGGTTCCTACTACTACTACTACATGGACGTC	3/4
			IGHV4-34/IGHD3-10/IGHJ6	GCGGGAACGGGGAGGCGCTACTACTACGGTATGGACGTC	1/4
	IgAN2-9	γ chain	IGHV4-59/IGHD3-22/IGHJ3	GCGGGGGATCGTTCATACTATGATAGCCGGAGTTTTTACCCTGATGCTCTTGATATC	5/5
		μ chain	IGHV3-53/IGHD6-19/IGHJ4	ACTGGAGATGATGGAACGTATAGCAGTGGCTGGTACCACGGCTAC	4/4
	IgAN2-10	γ chain	IGHV4-59/IGHD3-22/IGHJ3	GCGAGGGATCGTTCATACTATGATAGCCGGAGTTTTTACCCTGATGCTCTTGATATC	5/5
IgAN3	IgAN3-1	γ chain	IGHV5-51/IGHD3-16/IGHJ4	GCGGGATGGAATGTTATGATTGGATTTTATACAGCTGACTAC	2/2
		μ chain	IGHV3-7/IGHD2-21/IGHJ4	GCGAGGAGTGATTGGGGGCCTGACTAC	3/8
			IGHV3-74/IGHD5-18/IGHJ4	GCAAGAGCCCCCCCGGGTAGTTTCACTGGATACCATTTCGACTAC	1/8
			IGHV4-61/IGHD2-21/IGHJ5	GCGAGAGCGGATTGTGGTCGTGCTGGCTGTTACACCCCATTTTACAACTGGTTCGACCCC	4/8
	IgAN3-2	γ chain	IGHV3-23/IGHD6-13/IGHJ1	GCGAAAGACCGTGGGCGAATAGGAGCACCTGGTACTCTCGTCGTCGCCGTGAAACCCTCTGA<br>ATACTTCCAACAT	6/6
IgAN4	IgAN4-1	γ chain	IGHV1-18/IGHD3-9/IGHJ6	GCGAGAGTCTTAGTGGTTCACGATCCTTTGACTGGTTACCACTACAGTATGGACGTC	5/5
	IgAN4-2	μ chain	IGHV3-23/IGHD3-3/IGHJ5	GCGAACATAATGTATTGGCAGCCTGAATCC	1/4
			IGHV3-7/IGHD3-3/IGHJ5	GCGAACATAATGTATTGGCAGCCTGAATCC	3/4
		γ chain	IGHV1-8/IGHD3-3/IGHJ5	GCGAGAGGCAACCGGGGCCAGCATACTGCGATTTTTGGAGTGGTTATCCTATGGTGGTTCGA<br>CCCC	5/5

Cases No.	Cell No.	Ig type	V_HDJ_H recombination	CDR3 sequence	Clones
MN1	MN1-1	γ chain	IGHV1-69/IGHD3-3/IGHJ4	GCGAGAGTTCGAGGGGGGCGTATAACGATTTTTGGAGGGCTTGAGTAC	2/5
			IGHV3-15/IGHD2-8/IGHJ5	ACCACAGGATATTGTACTGATATTAGGTGCGTCACC	3/5
		μ chain	IGHV1-18/IGHD5-12/IGHJ5	GCGAGCACCAATAGTGGCTACGATGGGGGGCTTGGTCCC	5/5
	MN1-2	γ chain	IGHV1-18/IGHD1-1/IGHJ4	GCGAGGGACAACTTTATTTCGCCCACTGAAGGTCCCTTTGACAAC	7/7
		μ chain	IGHV1-18/IGHD6-19/IGHJ4	GCGAGAGACCTGGTTCGCGAAATAGCAGTGGCTGGTACGACGAGGAGGGGGGCTTTGACTAC	2/2
	MN1-3	γ chain	IGHV1-18/IGHD3-3/IGHJ6	GCGAGAGTCTTACAGGTTCACGATCCCTTGAGTGGTTACCACTGCAATATGGACGTC	10/10
	MN1-4	α chain	IGHV4-34/IGHD3-3/IGHJ1	GCGAGTCCAGACGGGAGTGCTTATGCCTTCCTCTAC	1/6
			IGHV3-7/IGHD3-10/IGHJ5	GTACTGTGGTTCGACATCGGAGGGAACCAC	5/6
		γ chain	IGHV1-46/N/A/IGHJ6	GCGAGATCCAAGAACGGAATGGACGTC	5/5
		μ chain	IGHV3-69/IGHD3-22/IGHJ5	GTGGGAGGATGCTATAGTAGTGGTTATTACTGGTTCGACCCC	6/6
	MN1-5	μ chain	IGHV3-48/IGHD2-15/IGHJ4	GCGCGAGATTTAGGATATTGTAGTGGTGGTCGCTGCTCCCTTATTTACCACTTTGACTCC	3/6
			IGHV4-34/IGHD6-13/IGHJ4	GCGAGGCTGTATAGCAGCAGCTGC	3/6
	MN1-6	γ chain	IGHV3-53/N/A/IGHJ6	GCGAGATCCATGGACGTC	8/8
		μ chain	IGHV1-3/IGHD6-25/IGHJ4	GCGACTGTAGCAGCGTCCTTGGGGGGCTAC	7/7
	MN1-7	μ chain	IGHV1-18/IGHD6-19/IGHJ4	GCGAGAGACTCAGAAATAGCAGTGGCTGGTACAGGCGACTAC	6/6
	MN1-8	μ chain	IGHV5-51/IGHD2-15/IGHJ6	GCGAGATATGGTCTGCGGGGCTGTAGTGATAAATGTTATGTTAGTTTCTACTACTACGGTATG GACGTC	4/4
MN2	MN2-1	γ chain	IGHV3-11/IGHD4-23/IGHJ6	GCGAGACTTCCTCGTGAGGTGGGAACTCCGTACTACTTCTACGGTATGGACGTC	5/5
		μ chain	IGHV3-15/IGHD6-13/IGHJ4	ACTTGGTCAAGCACCTGGTCACTCAGGTTCTACTTTGACTCC	4/8
			IGHV3-64/IGHD5-12/IGHJ4	GCGAGAGGGGGCCGGGTGGTGGCTACGGAGGCCGGCTAC	2/8
			IGHV4-59/IGHD2-21/IGHJ4	GCGAGAGGGGGGGAAGTGGTGACGACTAGCCCCTCTCACTTTGACTAC	1/8
			IGHV1-18/IGHD2-2/IGHJ4	GCGAGAGACGACGCGGATGGGTTGGACGAATCAGCTTCAATTGACTAC	1/8

Cases No.	Cell No.	Ig type	V_HDJ_H recombination	CDR3 sequence	Clones
MN2	MN2-2	μ chain	IGHV1-18/IGHD3-22/IGHJ4	GCGAGAACCCTTAGGCGTGAGAGTAGTGGTTATTCCCTATAC	6/6
	MN2-3	α chain	IGHV3-23/IGHD3-16/IGHJ4	GTGAAATATGGAGATTTCCTTTGGGGGGTTATCGTTTGAACTACTTTGACTAC	6/6
		μ chain	IGHV3-21/IGHD3-10/IGHJ4	GCGAGGTATTACTATGGTTCGTGGACTTATTATAACACCTACTTTGACTAT	4/4
	MN2-4	μ chain	IGHV1-18/IGHD1-26/IGHJ5	GCGAGAGATCGGAGTGGGAGCTACCCCCCCGCTTGGTGGTTCGACCCC	1/6
			IGHV3-23/IGHD3-10/IGHJ4	GCGAAAGGAGGGTATTACCATGGTTCGGGGAACTACTTTGACTTC	5/6
	MN2-5	μ chain	IGHV1-18/IGHD3-22/IGHJ4	GCAAGACTTCAGAGTGATAGTAGTGGTTATTACCACCAACCT	6/6
	MN2-6	μ chain	IGHV3-9/IGHD5-24/IGHJ4	GCAAAAGTGGGGTATGGCTACAATTACTGGGGGCACTTTGACTAC	5/5
	MN2-7	μ chain	IGHV1-18/IGHD3-22/IGHJ4	GCGAGAGATCATGATAGTAGTGGTTATTACGAACGGTTCTTTGACTAC	4/6
			IGHV1-58/IGHD2-15/IGHJ6	GTGGCAGGACCATATTGTAGTGGTGGTAGCTACTACGACTCCAATTACTACTACTACTACGGTAT GGACGTC	2/6
	MN2-8	γ chain	IGHV1-2/IGHD1-1/IGHJ5	GCGAGAGACCCTGAAGCGGGTTTGGAAGCAACTGATAACTGGTTCGACCCC	3/3
		μ chain	IGHV3-15/IGHD3-16/IGHJ4	GTTTGGTCAAGCACATGGTCAACTCGGTTCTACCTTGACCGC	5/6
			IGHV4-30/IGHD6-13/IGHJ4	GCGCTAGCGGAAGGCAGCAGCTGGTATCTCTACGACTAC	1/6
MN3	MN3-1	α chain	IGHV3-23/IGHD3-10/IGHJ3	GCGAGAGTCACGGAGTTTCACACTCCGCCCTCCTTTCGGGGGGATGCTTTTGATCTC	14/14
		γ chain	IGHV1-2/IGHD3-16/IGHJ6	GCGAGAGGGGGTCATAGGTATGATGATTACTATTACTACTACGGTATGGACGTC	12/12
	MN3-2	γ chain	IGHV1-69/IGHD2-15/IGHJ4	GCGACCCTAAAGGGATATTGCGGCTTTGGAAGCTGCTACTCGTTCTTTCTTGACGGCCCTGACAAC	10/10
IN	IN-1	μ chain	IGHV4-59/IGHD3-22/IGHJ3	GCGAGAGATGTAACGTATGATAGTAGTGGTTATAATGCTTTTGATATC	5/5
	IN-2	ε chain	IGHV3-23/IGHD2-21/IGHJ4	GCGCGAAGGGTGACGGACTTGGGGATCCGTCAG	5/5
	IN-3	ε chain	IGHV4-59/IGHD3-22/IGHJ3	GCGAGGGATCGTTCATACTATGATAGCCGGAGTCTTTACCCTGATGCTCTTGATATC	1/6
			IGHV3-30/IGHD2-2/IGHJ6	GCGAAAACGAAGGGTTGTAGTCGTACCACTTGCTACTATTACGGAATGGACATC	5/6

Cases No.	Cell No.	Ig type	V_HDJ_H recombination	CDR3 sequence	Clones
IN	IN-4	γ chain	IGHV3-9/IGHD3-10/IGHJ4	GCAAAAGATCTCGCCCCAACGGGGATGCTTCGGGGGCCATTTGATAAC	4/4
		μ chain	IGHV3-7/IGHD1-26/IGHJ6	GCGAGAATCGGGGGTGGGAGGTACGATGGGTACTACTACGGTCTGGACGTC	9/11
			IGHV1-18/IGHD5-12/IGHJ4	GCGAGATCCCTCTCATATGGTGGCTACGATTTCGCTTAC	2/11
		ε chain	IGHV3-9/IGHD3-10/IGHJ4	GCAAAAGATCTCGCCCCAACGGGGATGCTTCGGGGGCCATTTGATAAC	8/8
	IN-5	α chain	IGHV4-59/IGHD5-21/IGHJ6	GCGAGAGATAGGCATTATGGTGGCTACGATCGGGACTACTACTACTACATGGACGTC	5/6
			IGHV3-30/IGHD3-10/IGHJ4	GCGAGAGCGTATTACTATGGTTCGGGGAGTTATTATTACTTTGACTAC	1/6
		μ chain	IGHV4-59/IGHD5-21/IGHJ6	GCGAGAGATAGGCATTATGGTGGCTACGATCGGGACTACTACTACTACATGGACGTC	1/3
			IGHV3-30/IGHD3-10/IGHJ4	GCGAGAGCGTATTACTATGGTTCGGGGAGTTATTATTACTTTGACTAC	1/3
			IGHV4-34/IGHD3-10/IGHJ6	GCGAGAGGGCTTGAAGGCGGGGCGCACTACTACTACATGGACGTC	1/3
		δ chain	IGHV4-59/IGHD5-21/IGHJ6	GCGAGAGATAGGCATTATGGTGGCTACGATCGGGACTACTACTACTACATGGACGTC	6/9
			IGHV4-34/IGHD3-10/IGHJ6	GCGAGAGGGCTTGAAGGCGGGGCGCACTACTACTACATGGACGTC	2/9
			IGHV3-30/IGHD3-10/IGHJ4	GCGAGAGCGTATTACTATGGTTCGGGGAGTTATTATTACTTTGACTAC	1/9
		ε chain	IGHV3-23/IGHD2-21/IGHJ4	GCGCGAAGGGTGACGGACTTGGGGATCCGTCAG	7/7
	IN-6	α chain	IGHV1-24/IGHD5-18/IGHJ1	TCTATTGGGGCTATGGCCACC	7/7
		γ chain	IGHV1-69/IGHD2-15/IGHJ4	GCGACCCTAAAGGGATATTGTGGCTTTGGAAGCTGCTACTCGTTCTTTCCTGACGGCCCTGACAAC	10/10
	IN-7	δ chain	IGHV4-34/IGHD2-2/IGHJ6	GCGAGAATTGTAGTAGTACCAGCTGCCTTCGGAAGAGGTTATTACTACTACTACTACGGTATGGAC GTC	5/5
	IN-8	γ chain	IGHV3-7/NA/IGHJ6	GTGGGGGCCTACGGTATGGACGTC	5/5
	IN-9	μ chain	IGHV4-34/IGHD3-22/IGHJ4	GCGAGAGGCCGGTCGTATGATAATAGTGGTTATCCAAACCCCTTGGACTAC	5/5
		ε chain	IGHV3-23/IGHD2-21/IGHJ4	GCGCGAAGGGTGACGGACTTGGGGATCCGTCAG	5/5
